# Quadrantic vortex vein decompression with subretinal fluid drainage for manangement of Nanophthalmic choroidal effusions— a review of literature and case series

**DOI:** 10.1186/s12886-019-1213-z

**Published:** 2019-10-24

**Authors:** Anadi Khatri, Sweta Singh, Kriti Joshi, Muna Kharel

**Affiliations:** 1 Ophthalmologist, Vitreo-Retinal Surgeon, Birat Eye Hospital, Biratnagar, Nepal; 2 Ophthalmologist, Vitreo-Retinal Surgeon, Lumbini Eye Institute and Research Center, Lumbini, Nepal; 3 Ophthalmologist, Lumbini Eye Institute and Research Center, Lumbini, Nepal; 4 Resident of Ophthalmology, Nepalese Army Insitute of Health Sciences, Kathmandu, Nepal

**Keywords:** Uveal effusion, Nanophthalmos, External drainage, Sclera, Exudative

## Abstract

**Background:**

Uveal effusion syndrome is a rare entity of idiopathic exudative detachments of uveal tissues and retina. Medical treatments with systemic steroids and antimetabolites have been tried but with variable results. Scleral windows or vortex decompressions are usually performed and surgeons usually perform partial sclerectomy in all the quadrants.

**Case presentation:**

For the first time, we report 2 cases of nanophthalmic uveal effusion syndrome managed with our technique.

**Conclusion:**

Quadrantic vortex vein decompression with external drainage for nanophthalmic uveal effusion can provide immediate and stable gain in vision.

## Introduction

Uveal effusion syndrome (UES) is a rare syndrome causing idiopathic exudative detachments of the choroid, ciliary body, and retina. It is hypothesized to be caused by impaired vascular drainage usually associated with scleral thickening [1]. The condition was first described by Brockhurst in 1975 but the concept has basically remained the same even today.

The thickened sclera is often a result of abnormal collagen fiber which compresses/impinge the vortex vein causing disturbances in venous drainage [2, 3].

Gass further added that most of the primary idiopathic uveal effusion syndrome are likely to be related with such congenital sclera anomaly - while in some cases, UES could also occur due to abnormal vortex veins. Various ocular pathologies or states like postoperative hypotony, scleral buckling surgery or posterior scleritis could also give rise to UES [1]. Whatsoever the pathology, they all share the final common pathway which will ultimately lead to congestions of the choriocapillaris and choroidal vasculature resulting in accumulation of fluid in the subretinal/subuveal space [6, 7].

Nanophthalmos is a rare primary ocular disorder that occurs due to arrest of growth of the globe after the closure of embryonic fissure. It is characterized by narrow palpebral fissure, small orbit, deep-set globe and a short axial length with no gross structural defect. These eyes are hyperopic with normal or reduced corneal diameter. It usually occurs bilaterally and sporadically, but can also occur as an inherited form in either autosomal dominant or recessive pattern [3, 4].

The examination of such eyes usually shows crowding of the anterior chamber structures –which is mainly due to a high lens - to - eye volume ratio. This is one of the reasons why nanophthalmic eyes have predeliction to angle-closure glaucoma and uveal effusion [5].

Nanophthalmos is classified into mainly three variants - Nanophthalmic eye or type 1. The eyeball is small (average axial length 16 mm) and high hypermetropic (average + 16 diopters). In type 2 or non-nanophthalmic eyes, there is clinically evident abnormal sclera but the eyeball size is normal (average axial length 21 mm) with small refractive error. The type 3 or non-nanophthalmic variant has clinically normal sclera [7, 9]. The natural course of UES in each of these variants is variable and often associated with frequent relapses.

Most nanophthalmic uveal effusion was left conservatively treated in the past. However, with the availability of new drugs and surgical breakthroughs over time, various methods of management have been described. Medical treatments with systemic steroids and antimetabolites have also been used but with variable and questionable results [2].

Surgical procedure gained momentum and popularity when Brockhurst in 1980, described good surgical results with decompression of the vortex veins by scleral resection with sclerotomy [8, 9].

In 1983, a newer surgical approach- subscleral sclerectomy was introduced by Gass and Jallow [1]. However, this technique had its limitations as it was later demonstrated that it was effective only in Types I and II nanopthalmic eyes [10]. With developments of newer devices, in 2015, Yepez JB et al. mentioned use of Ex-PRESS shunt, a glaucoma implant (2- to 3-mm–long and 0.4-mm–diameter tube). They reported successful treatment of type 2 uveal effusion syndrome. The device shunted the aqueous humor from the anterior chamber into the subconjunctival space and lowered the IOP [11].

Despite all the advances and techniques, the surgery on nanophthalmic eyes is very unpredictable. But if left untreated, most of these patients usually tend to get worse with time [12]. Hence the dilemma to intervene or not exists even today. As no generalized consensus is available, the treatment option is advised to be kept as “individualized treatment” with anticipation of all the complications that may pursue.

Scleral windows or vortex decompressions are usually performed and surgeons usually perform partial sclerectomy in all the quadrants in these procedures. It is the surgeon’s preference of whether they want to perform the external drainage of subretinal fluid or not. Here we describe, 2 patients of UES due to nanophthalmos for whom we performed quadrantic (only two quadrants) scleral window surgery (vortex vein decompression) with subretinal drainage at a single setup and their outcomes.

## Case presentation

### Case 1

A 32-year-old man was referred to our retina clinic due to decreased visual acuity in his left eye since 6 years. He had been wearing glasses for hyperopia since the age of 12. A gradual decrease in visual acuity in both his eyes had led him to seek care at multiple eye centers in the last 2 years. He was diagnosed with serous retinal detachment in his left eye and was referred to our clinic.

**Fig. 1:**
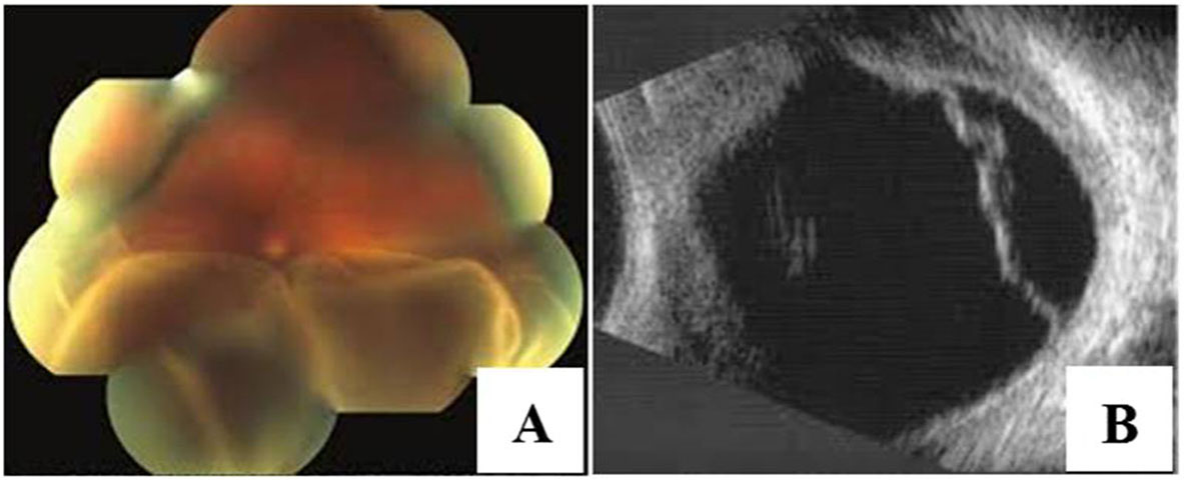
Exudative retinal detachment with choroidal effusion in the nanophthalmic eye. (Preoperative finding) **a**. Fundus Photo sowing exudative retinal detachment. **b** USG scan of the same eye

**Fig. 2:**
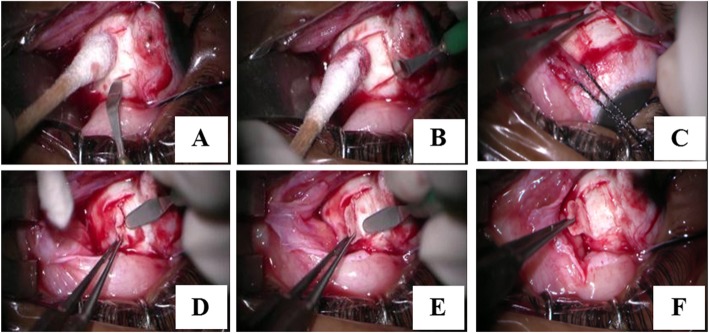
Surgical technique of the quadrantic scelerectomy. After peritomy and muscle isolation, a 5 × 7 mm scleral tissue inbetween the insertion of two recti are marked (**a**, **b**, **c**). A cresent is used to first mark all the borders in a lamellar fashion and then lamellar sclerectomy is perfomed (depth 2/3rd to 4/5th). (**d**, **e**, **f**) Same in performed in one more another quadrant. Quadrant can be used as per surgeons preference but we used supero temporal and inferotemporal in both our cases

**Fig. 3:**
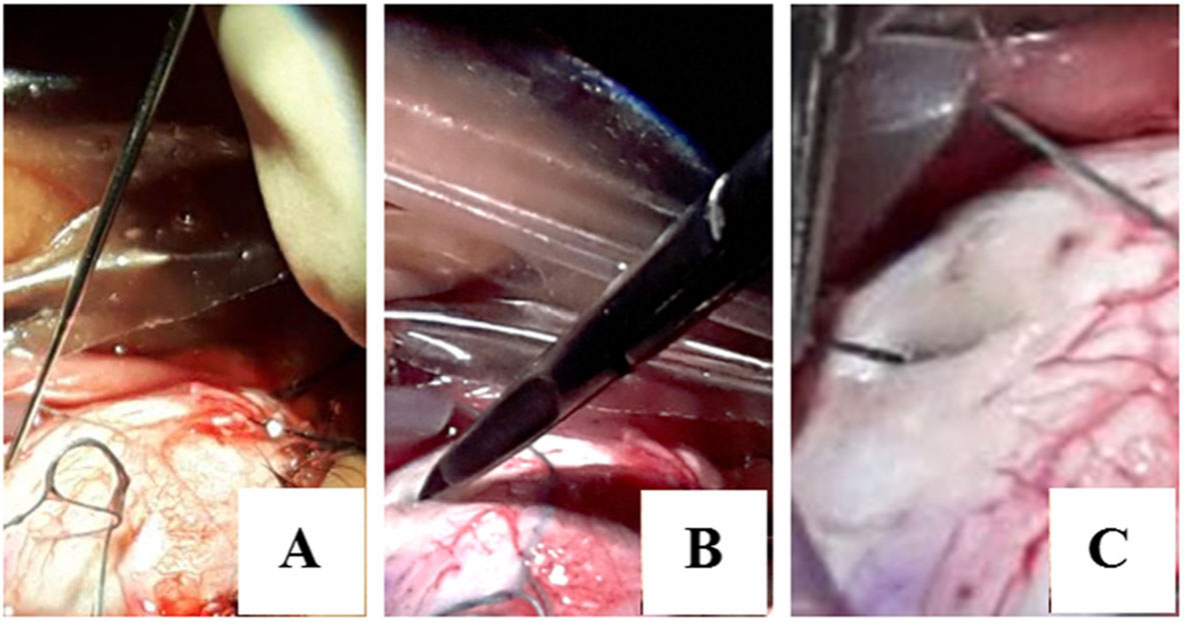
Cut down technique for performing external drainage. The sclera is cauterized in the area where drainage is intended (**a**). No 13 surgical blade is used to cut down the sclera in layer by layer fashion until choroidal hue is present (**b**). The bed is cauterized and then a 10–0 needle or a 27G needle is used to perforate the choroid to reach the subretinal space and drain the fluid externally (**c**)

At the time of his presentation, his visual acuity was 6/24 in the right eye and 2/60 in his left eye. Best-corrected visual acuity (BCVA) was 6/6 and 6/60 with spherical equivalents of + 6.00 and + 11.5 diopters (D) in the right and left the eye, respectively. Fundus examination revealed leopard-spotted retinal pigment epithelial changes, macular hypoplasia, and crowded disc features. In the left eye, the presence of serous retinal detachment involving the macula was noted. The axial length was 21.04 mm in the right and 18.9 mm in the left eye on A-scan and B-scan ultra-sonograms (Fig. 1). Optical coherence tomography (OCT) showed a thickened sclera 1.58 mm and 1.72 mm in the paralimbal region of right and left eye respectively. Surgical management was planned for the left eye. Under peribulbar anesthesia, a vortex vein decompression was performed using ~ 80% of the scleral thickness – flaps measuring 5 mm by 7 mm in two quadrants of the left eye-inferotemporal and superotemporal (Fig. 2). An anterior chamber maintainer was placed via clear corneal incision in a quadrant where it would least disturb the eye movement or expel itself -often corresponding to 1 clock hour inferior to the region of drainage. Subretinal fluid was drained via cut down technique externally (Fig. 3).

The patient was started on acetazolamide tablets three times a day along with oral steroids of 1 mg /kg, indomethacin sustained-release capsules 75 mg twice daily and proton pump inhibitors. The eye was kept padded until the next day.

**Fig. 4:**
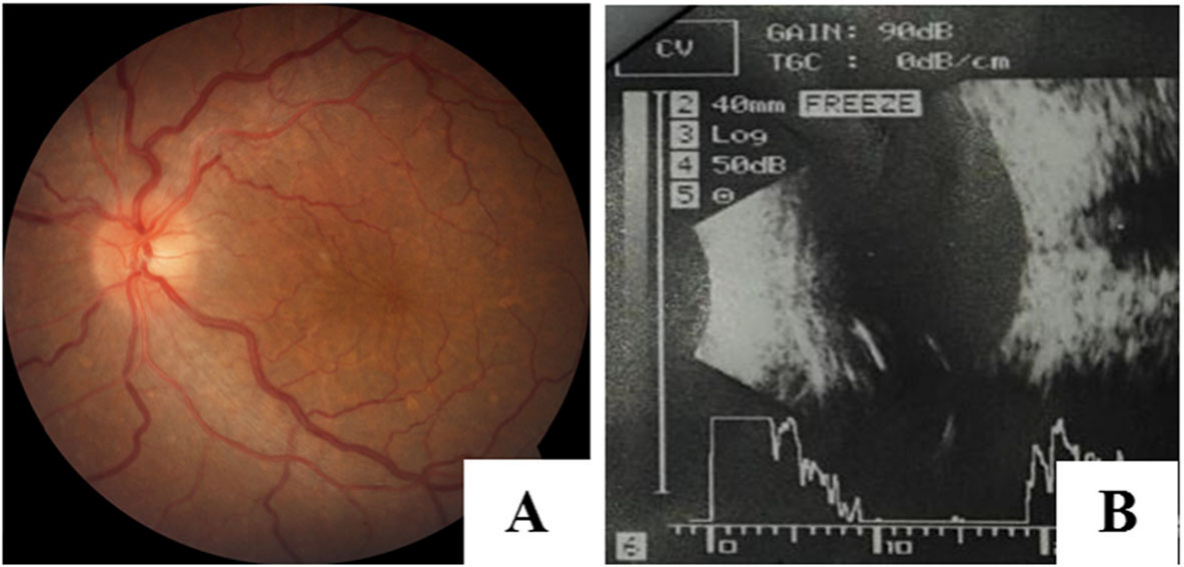
Fundus photo (**a**) and B scan (**b**) of the attached retina on the first post operative day

On the first postoperative day, a flat retina was seen on fundus examination (Fig. 4), with a BCVA of 5/60. Oral acetazolamide was advised to be continued for 3 days and steroids tapered off on a weekly basis until 4 weeks. Topical steroids and antibiotics drops were prescribed and were continued for a total period of 6 weeks. The retina remained attached during the follow-up period, and BCVA of 6/18 with + 7 diopter was achieved at 8 weeks.

### Case 2

**Fig. 5:**
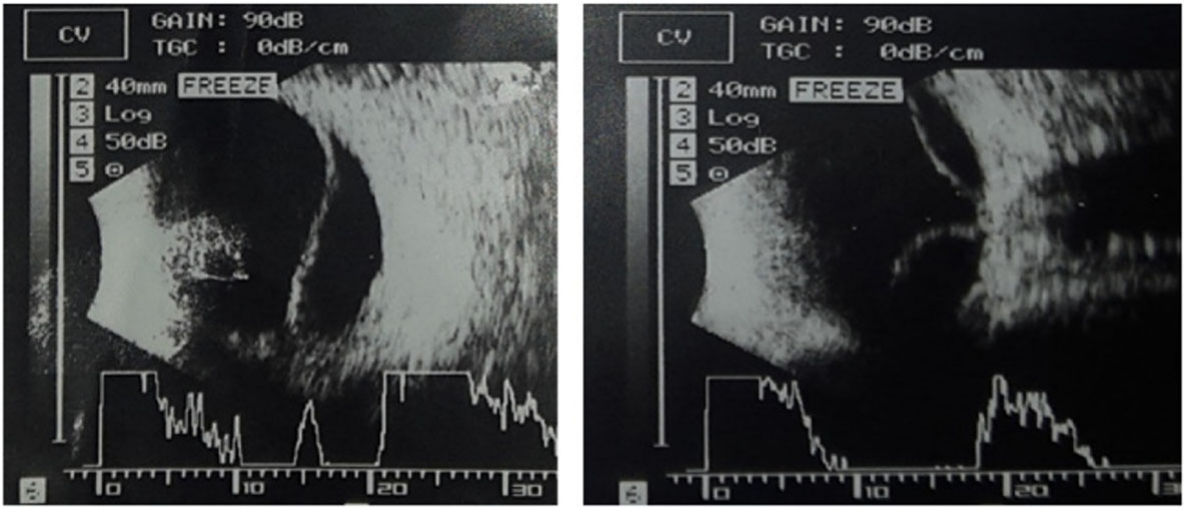
Retinal Detachment with choroidal thickening suggestive of choroidal effusion

A 45 years old female with decreased vision in the left eye for 4 years presented with BCVA was 6/24 in the right eye with +13D and 2/60 in the left eye. Fundus examination revealed mottled retinal appearance and crowded disc features. Serous retinal detachment was noted in the left eye with involvement of the macula. The intraocular pressure was 22 mmHg and 20 mmHg in right and left eye respectively. The axial length was 14.4 mm in the right eye and 15.7 mm in the left eye on A-scan and B-scan ultrasonograms. The anterior segment was normal but an examination of the posterior segment showed a total retinal and choroidal detachment in the left eye. This was further confirmed using ultrasound (Fig. 5).

**Fig. 6:**
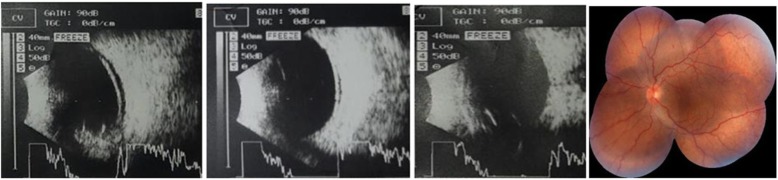
Resolution of the subreitnal fluid in subsequent visits and complete resolution by the 12th week of followup

The scleral thicknesses measured by OCT were 1.92 mm and 1.70 mm, respectively in right and left the eye. Left eye surgical decompression of vortex vein and drainage of subretinal fluid was planned and performed subsequently. The vortex veins were decompressed again at supero temporal and supero inferior quadrant, followed by placement of anterior chamber maintainer and drainage of the subretinal fluid externally in the same setting. The post-operative medications were the same as described in case 1. After the surgery, BCVA was 3/60, 5/60 with + 9.50 diopter and 6/36 with + 9 diopter on the 1st postoperative day, 1st week after the operation and on the 12th week of operation respectively. The remaining subretinal fluid resolved gradually postoperatively and resolved completely at the 12th week of follow-up (Fig. 6).

## Discussion and conclusions

In 1963, Schepens and Brockhurst first reported uveal effusion syndrome (UES) in which they described the pathology as choroidal detachment, often with secondary retinal detachment, optic disc swelling, and minimal signs of uveitis [13].

Several theories have been speculated on UES pathophysiology, including obstruction of the vortex vein, increased choroidal permeability, intrinsic choroidal changes, and decreased scleral permeability [4, 14]. Moreover, Forrester et al. [15] has observed the migration of retinal pigment epithelial cells into the subretinal space in cases with uveal effusion syndrome giving rise to the “leopard spot” hyperpigmentation [2]. A type of uveal effusion syndrome comes in association with nanophthalmic fundus and such eyes mostly present with the axial length of the upper limit of < 20 mm.

We have reported two cases of nanophthalmic uveal effusion that were treated surgically with quadrantic vortex vein decompression with subretinal fluid drainage. In both cases, there was significant anatomical improvement in immediate postoperative period with gradual functional recovery with no significant complication noted.

Uyama et al. treated 19 eyes of 16 patients with UES by making a two-third–thickness scleral flap and performing a scleral excision to expose the underlying choroid. Miranda et al. [16] reported a case of type 1 UES with bilateral retinal detachment and undergone two- thirds thickness sclerectomy (4 mm × 5 mm) (our Dimensions 5 mm × 7 mm) and sclerotomy procedure, at the equator of the inferotemporal and inferonasal quadrants showing a slow but significant improvement in the subretinal fluid resolution. Six months postoperatively, the choroidal effusions disappeared and VA improved to 20/100. Results were similar to ours, however anatomical and functional recovery was achieved quicker in our cases.

In contrary, Jong et al. [6] reported a case of young boy of 13 years old with nanophthalmic uveal effusion. He was kept under observation without any medication and his clinical findings were unaltered for years. M Kong et al. [7] mentioned a case of nanophthalmic UES, who underwent drainage sclerotomy in all four quadrants of the eye. Here, unlike in our case, a flat retina was seen on fundus examination and OCT, only at 1 month follow up.

Our method of Quadrantic vortex vein decompression with subretinal fluid drainage at the same setting can be advantageous in treatment for nanophthalmic uveal effusion syndrome with early and stable visual recovery.

### Limitation

Small sample size is a limitation in this study.

## Data Availability

Not Applicable.

## Abbreviations

BCVA: Best-corrected visual acuity
D: Diopters
OCT: Optical coherence tomography
UES: Uveal effusion syndrome
